# Different Relationship between *hsp70* mRNA and hsp70 Levels in the Heat Shock Response of Two Salmonids with Dissimilar Temperature Preference

**DOI:** 10.3389/fphys.2016.00511

**Published:** 2016-11-07

**Authors:** Mario Lewis, Miriam Götting, Katja Anttila, Mirella Kanerva, Jenni M. Prokkola, Eila Seppänen, Irma Kolari, Mikko Nikinmaa

**Affiliations:** ^1^Laboratory of Animal Physiology, Department of Biology, University of TurkuTurku, Finland; ^2^Natural Resources Institute Finland (Luke)Enonkoski, Finland

**Keywords:** heat shock response, heat shock proteins, salmonids, climate change, chaperones, temperature acclimation

## Abstract

The heat shock response (HSR) refers to the rapid production of heat shock proteins (hsps) in response to a sudden increase in temperature. Its regulation by heat shock factors is a good example of how gene expression is transcriptionally regulated by environmental stresses. In contrast, little is known about post-transcriptional regulation of the response. The heat shock response is often used to characterize the temperature tolerance of species with the rationale that whenever the response sets on, a species is approaching its lethal temperature. It has commonly been considered that an increase in *hsp* mRNA gives an accurate indication that the same happens to the protein level, but this need not be the case. With climate change, understanding the effects of temperature on gene expression of especially polar organisms has become imperative to evaluate how both biodiversity and commercially important species respond, since temperature increases are expected to be largest in polar areas. Here we studied the HSR of two phylogenetically related Arctic species, which differ in their temperature tolerance with Arctic charr having lower maximally tolerated temperature than Atlantic salmon. Arctic charr acclimated to 15°C and exposed to 7°C temperature increase for 30 min showed both an increase in *hsp70* mRNA and hsp70 whereas in salmon only *hsp70* mRNA increased. Our results indicate that the temperature for transcriptional induction of *hsp* can be different from the one required for a measurable change in inducible hsp level. The species with lower temperature tolerance, Arctic charr, are experiencing temperature stress already at the higher acclimation temperature, 15°C, as their *hsp70* mRNA and hsp70 levels were higher, and they grow less than fish at 8°C (whereas for salmon the opposite is true). Consequently, charr experience more drastic heat shock than salmon. Although further studies are needed to establish the temperature range and length of exposure where *hsp* mRNA and hsp level are disconnected, the observation suggests that by measuring both *hsp* mRNA and hsp level, one can evaluate if a species is approaching the higher end of its temperature tolerance, and thus evaluate the vulnerability of an organism to the challenges imposed by elevated water temperature.

## Introduction

The regulation of heat shock protein expression is one of the most studied systems of gene expression, and the function and induction of heat shock proteins has been reviewed in detail from both basic and comparative angle (Lindquist and Craig, 1988; Feder and Hofmann, 1999; Basu et al., 2002; Richter et al., 2010; Deane and Woo, 2011). Especially the transcriptional induction of heat shock genes has been fully characterized, and the role of heat shock factors—prototypes of transcriptional activators—in the response has been detailed (Lindquist, 1986; Morimoto, 1993; Sistonen et al., 1994; Prahlad and Morimoto, 2009). In comparison, post-transcriptional regulation of heat shock protein production has been little studied (Silver and Noble, 2012), although it is clear that it also contributes to the hsp level after rapid temperature increase (Theodorakis and Morimoto, 1987). Particularly the stability of mRNAs of genes encoding inducible heat shock proteins appears very temperature-sensitive (Theodorakis and Morimoto, 1987).

On the basis of available literature, post-transcriptional regulation of the heat shock response plays a role in hsp accumulation in vertebrates (Silver and Noble, 2012), e.g., at the high pressure experienced by chondrocytes (Kaarniranta et al., 1998) and in exercise adaptation (Melling et al., 2007). Further, differences between cell types with regard to post-transcriptional regulation of the HSR in mammals have been reported (Kaarniranta et al., 2002). In *Xenopus* oocytes heat shock protein production is completely translationally regulated: upon adequate increase in temperature, repression of heat shock protein production is released, and premade mRNA is translated to heat shock protein (Bienz and Gurdon, 1982). Uncoupling of the transcription of genes encoding heat shock proteins and the actual protein production has not been much studied in fish. However, two studies have shown that such disconnection of mRNA and protein production may take place. First, Lund et al. (2002) have observed that in salmon a higher temperature seems to be required for inducible heat shock protein production than for the induction of mRNA production from the *hsp* gene. Second, Hofmann et al. (2005), studying two New Zealand notothenioids *Bovichtus variegatus* Richardson, 1846 and *Notothenia angustata* Hutton, 1875, showed that in the former species both the hsp and *hsp* mRNA production increased in heat shock, whereas in the latter only *hsp* mRNA production increased. Further, even in *B. variegatus* the temperature for *hsp* protein and mRNA induction may have been different.

Thorough understanding of the regulation of the heat shock response in aquatic poikilotherms has become imperative with climate change, since the temperature responses of a species will affect its capability to acclimate to warming water. Also, finding responses which change with small temperature increase are most valuable, as they can show a perturbation in the living conditions of a species with likely occurring near-future conditions. Already earlier it has become clear that acclimation to different temperatures affects both the temperature where the heat shock response is induced and where it is maximal (Dietz and Somero, 1992), and that phylogenetically related organisms inhabiting different temperatures (e.g., in different tidal zones in the same area) exhibit different induction temperatures (Podrabsky and Somero, 2004; Tomanek, 2010). We have studied the heat shock response using two phylogenetically related salmonids, the Arctic charr (*Salvelinus alpinus*) and Atlantic salmon (*Salmo salar* m. *sebago*) with overlapping distributions. The populations used in our study originate from the same lake area. Both species inhabit Arctic areas, where temperature increase has been greatest in the recent past (e.g., Belkin, 2004; Wanishsakpong et al., 2016). Thus, the distribution of these fish can be drastically affected by climate change. Notably, the Arctic charr has become an important aquaculture species, but starts to suffer if the rearing temperature exceeds 14°C (Quinn et al., 2011). The acute tolerance of Atlantic salmon and Arctic charr to temperature change (as measured by the loss of equilibrium with increased temperature) is different with lower temperatures tolerated by charr (Anttila et al., 2015).

We studied the inducible *hsp70* gene, particularly the one for which a specific antibody is commercially available, as it has been commonly used in temperature studies of salmonids (e.g., Lund et al., 2002), and since *hsp70* mRNAs were earlier shown to increase most when Arctic charr were exposed to 15–19°C (Quinn et al., 2011). We hypothesized that the levels of *hsp* mRNAs and proteins after acclimation to 8 and 15°C for a month and the heat shock responses of Arctic charr and Atlantic salmon are different. We focussed especially on the question, if the induction of protein and mRNA production of the *hsp70* gene can occur at different temperatures and be different in the two species. This was done especially, since, although it is known that the mRNA and protein production of the genes are often uncoupled (e.g., Jayapal et al., 2008; Logan and Buckley, 2015), it is commonly considered that in the case of heat shock proteins determining only the mRNA level suffices to conclude that also the protein level has increased (e.g., Deane and Woo, 2011) despite the information that disconnection between the two may occur (Lund et al., 2002; Hofmann et al., 2005). We further predicted that the differences between the species can be related to their earlier determined temperature tolerance. As a consequence, the study forms a basis of further investigations establishing the utility of heat shock response components in determining the position of a salmonid in its thermal tolerance window. Earlier, the interactions between thermal tolerance and heat shock response components in fish have mainly been studied with *Fundulus heteroclitus* (e.g., Healy et al., 2010), and, for example, diurnal variations in the increase of mRNA level after a slight heat shock have been observed (Healy and Schulte, 2012).

## Material and methods

### Experimental animals, acclimation and heat shock procedure

The experiments were conducted at the Natural Resources Institute Finland in Enonkoski, eastern Finland, from 1st July to 10th August 2013. All procedures were approved by the Finnish Animal Experiment Board (ESAVI/4068/04.10.07/2013). Arctic charr and Atlantic salmon originated from Lake Saimaa (62°04′ N; 28°33′ E) and were reared under a natural photoperiod at the Natural Resources Institute Finland hatchery for 3 and 1 generations, respectively. Juvenile (~1-year-old) charr and salmon were kept separately in 320 L cylindrical (90 cm diameter) tanks with constantly flowing, filtered, aerated, and temperature-controlled water from Lake Pahkajärvi. A 100 fish per tank of each species were acclimated to either 8°C (body mass 26.6 ± 1.3 g and fork length 14.5 ± 0.2 cm for charr and 22.8 ± 0.6 g and 12.8 ± 0.1 cm for salmon; mean ± SEM. at the end of acclimation) or 15°C (22.9 ± 1 g and 13.8 ± 0.2 cm for charr, and 27.5 ± 1 g and 13.6 ± 0.2 cm for salmon) for 4 weeks and fed commercial fish pellets (Raisio Group, Finland) *ad libitum*. The 4-week acclimation period was considered to be adequate for any acclimation responses to take place, and is also close to the longest period of time that the temperature can be expected to remain constant in nature. The photoperiod was ~17:7 L:D during sampling. Feeding was stopped 24 h prior to sampling and fish were sacrificed in 200 ppm tricaine methanesulfonate (MS-222, Sigma-Aldrich USA) buffered with sodium bicarbonate. Fish mass and fork length were measured before gills and liver tissue were excised and immediately frozen in liquid nitrogen. Forty fish per acclimation group were used for obtaining undisturbed values, and organs were taken at 1, 8, 16, and 21 h after the start of the light period, whereby the last sample was taken in the dark period. The *hsp70* mRNA and protein values were determined from 7 organs at every time point. The remaining 60 fish were subjected to a non-lethal heat shock. The shock was of exactly the same magnitude at both temperatures and for both species. This actually makes the heat shock more robust for Arctic charr than for Atlantic salmon, since the CTmax of charr is 1–2°C lower for charr than for salmon: 26.7 ± 0.07°C and 27.6 ± 0.07°C (SEM) in 8°C acclimated charr and salmon, respectively; 28.0 ± 0.07°C and 29.8 ± 0.08°C in 15°C-acclimated charr and salmon, respectively (Anttila et al., 2015), Water temperature was controlled using a 2 kW water heater (RC20 WGW Lauda, Germany). Submersible air-pumps and water-pumps were used to maintain oxygen saturation and prevent stratification of water temperature, respectively. Because handling has been shown not to affect the heat shock response (Vijayan et al., 1997), at the start of the light period the fish from each acclimation group were transferred to an experimental tank with water temperature 7°C higher than the acclimation temperature. Fish were kept at the heat shock temperature for 30 min before being returned to the acclimation tanks for recovery. The length of the heat shock and follow-up period were chosen arbitrarily, but it was checked that they were adequate to see a response both at mRNA and protein level in charr. Since it is probable that the ultimate signal of hsp production is the amount of misfolded protein, the magnitude of the response will be affected by the initial temperature, the temperature change in the heat shock, and the length of the exposure to increased temperature. Gills and liver tissue (chosen to represent two different tissues, one in direct contact with the environment and the other being metabolically a very active one) were subsequently excised at 1, 2, 4, 8, 16, and 24 h post-heat shock and flash frozen in liquid nitrogen for downstream analyses. We determined the mRNA levels using quantitative real time PCR and protein levels with western blotting from 7 fish per time point.

### Gene cloning, sequence validation and primer design

Primers used to amplify salmonid *inducible hsp70* were designed based on alignments of several salmonid *hsp70* mRNA sequences available at NCBI (www.ncbi.nlm.nih.gov), with accession numbers NM_001124228, NM_001124745 and AB062281.1 (*Oncorhynchus mykiss*), KF783199.1 (*Salvelinus fontinalis*), AJ632154.1 (*Salmo salar*) and OTU35064 (*Oncorhynchus tschawytscha*). Primers used to amplify an 812 base-pair (bp) gene fragment of *hsp70* in both charr and salmon are: For—CCTCTACATTCATAAACTGCAACT, Rev—CTGGCTGATGTCCTTCTTGTGT. To ensure that only the *inducible hsp70* isoform is amplified, a region with sufficient mismatch base-pairings with *S. salar hsc70* (BT059361) was selected for qPCR primer design.

Primers for β*-actin* were designed based on mRNA sequences with accession numbers AB196465.1 (*O. mykiss*), AB111057.1 (*Oncorhynchus nerka*), JR540730.1 (*Salvelinus alpinus*) and NM_001123525.1 (*S. salar*). Primers used to amplify a 1128 bp gene fragment of β*-actin* in both species are: For—ATGGAAGATGAAATCGCCGCAC, Rev—TTAGAAGCATTTACGGTGGACG. PCR products were obtained from cDNA reverse transcribed from 1 μg total RNA extracted from both species. RNA isolation and cDNA synthesis methodology is detailed in the succeeding section. Amplification of the gene of interest and reference gene was performed using a KAPA HiFi HotStart PCR Kit (KAPA Biosystems, USA) with the following thermal cycling parameters: 1 cycle of initial denaturation for 3 min at 95°C, then 30 cycles each of second denaturation at 98°C for 20 s, annealing at 60°C for 15 s and extension at 72°C for 60 s/kb. PCR products were size separated by electrophoresis in 1.5% agarose gel stained with ethidium bromide, followed by gel extraction using a NucleoSpin gel and PCR clean up kit (Macherey-Nagel, Germany).

Gene fragments were ligated onto a pJET1.2/blunt cloning vector with a CloneJet PCR Cloning kit (ThermoScientific, USA), propagated in CaCl_2_ competent DH5α *E. coli* and screened on LB-agar containing ampicillin. Positive colonies were selected for further propagation then purified with a NucleoSpin Plasmid EasyPure Kit (Macherey-Nagel, Germany). Sequencing was performed on purified plasmids at the European Custom Sequencing Centre (GATC Biotech AG, Köln Germany) and obtained sequences (*Hsp70*—KU885452 for *S. alpinus* and KU885451 for *S. salar;* β*-actin*—KU885450 for *S. alpinus* and KU885449 for *S. salar*) were aligned and confirmed with homologous sequences using NCBI BLAST. Phylogenetic analysis of the *hsp70* sequences from charr and salmon, done according to Metzger et al. (2016), confirmed that the cloned genes belong to the inducible *hsp70* isoforms, but our analysis could not differentiate between *hsp70-1* and *hsp70-2*. Species and gene-specific Taqman qPCR primers and fluorescence probes were designed using the Universal Probe Library Assay Design Centre website (Roche Diagnostics). Taqman primers (*hsp70* For—AGCTAAAGGCCCGTCTATCG, Rev—AACACCCCCACACAGGAGTA, Probe # 104 cat. no. 04692225001; Roche Diagnostics); β*-actin* For—CCAAAGCCAACAGGGAGA, Rev—GTACATGGCAGGGGTGTTG for charr and Rev—GTACATGGCGGGGGTGTTG for salmon, Probe # 115 cat.no. 04693493001; Roche Diagnostics) were designed to amplify a 60–65 bp amplicon and a further alignment of probe # 104 with *S. salar hsc70* was conducted to confirm that the probe did not bind to the transcripts of the constitutively expressed isoform. All primers were tested for efficiency and amplification signals obtained were within the quantifiable range of primer efficiencies (90–110%).

### Quantitative real-time PCR procedure for *hsp70* mRNA determination

Total RNA was extracted from tissues using the guanidine isothiocyanate method (Chomczynski and Sacchi, 1987) with TRI Reagent (Molecular Research Centre, USA), according to the manufacturer's instructions with additional purification steps. Frozen tissues were placed in TRI Reagent and homogenized mechanically with a TissueLyser (Qiagen, USA) at 30 shakes/s for 2 min. Phase separation of RNA was performed using 1-bromo-3-chloropropane, followed by isopropanol precipitation, washing with 75% ethanol, then the RNA was dissolved in RNase free water. To remove residual genomic DNA contamination, DNase I (Promega, USA) was added (1 μg) in solution to an aliquot of RNA and incubated for 10 min at 37°C, followed by another round of phase separation, precipitation and washing. The purified RNA was stored overnight at +4°C in 75% ethanol to ensure the thorough removal of potential contaminants, then centrifuged at 7500 RCF for 5 min, subsequently air-dried and re-dissolved in RNase free water. RNA concentration and purity were measured using a Nanodrop 2000 spectrophotometer (ThermoScientific, USA). Only samples with an A260/280 ratio of ≥1.8 were used in downstream applications.

RNA integrity was confirmed by agarose gel electrophoresis using sodium hypochlorite as a denaturant, as described previously (Aranda et al., 2012). An aliquot of RNA (600 ng) from each sample was mixed with 10X loading buffer (1.9 mM xylene cyanol, 1.5 mM bromophenol blue, 25% glycerol) and pipetted onto a gel comprised of 1% agarose, 1% commercial bleach (Kiilto, Finland) containing 6% sodium hypochlorite and stained with ethidium bromide. To test for genomic contamination, qPCR was performed without reverse transcription on each RNA sample in triplicate in a final reaction volume of 10 μl per well, including 2 ng of RNA, 0.3 μM of *hsp70* forward and reverse primers, 0.1 μM of probe # 104 and 5 μl 2X KAPA Probe Fast qPCR kit master mix (KAPA Biosystems, USA). Thermal cycling parameters are the same as in the qPCR methodology detailed in the succeeding section. Samples which did not amplify after 40 cycles were deemed free of genomic DNA and samples which amplified were re-treated with DNase I and purified as described above.

An aliquot of RNA (100 ng) from each sample was used for cDNA synthesis using a PTC-150 MiniCycler (MJ Research, USA), with a DyNAmo cDNA synthesis kit (ThermoScientific, USA) according to the manufacturer's instructions, in a final reaction volume of 20 μl inclusive of random hexamers, reverse transcription buffer with dNTP mix and MgCl_2_, M-MuLV RNase H+ reverse transcriptase and the following thermal cycling parameters: Primer extension at 25°C for 10 min, cDNA synthesis at 37°C for 1 h and reaction termination at 85°C for 5 min. Resultant cDNAs were subsequently stored at −20°C. qPCR was conducted using a 7900HT Fast Real-Time PCR System (Applied Biosystems, USA) for the undisturbed data and QuantStudio 12K Flex Real Time PCR System (Applied Biosystems, USA) for the heat shock data, in a final reaction volume of 10 μl, with 1 ng of cDNA, 0.3 μM forward and reverse primers, 0.1 μM probe and 5 μl 2X KAPA Probe Fast qPCR master mix (KAPA Biosystems, USA), with the following thermal cycling parameters: Stage 1 (enzyme activation) at 50°C for 2 min. Stage 2 (denaturation) at 95°C for 10 min, and 40 cycles of Stage 3 at 95°C for 15 s, then 60°C for 1 min (annealing and extension). Temperature changes were kept at a constant 1.6°C/s. Target and reference gene reaction quantities were determined from a standard curve generated from a 1:2 (undisturbed) and a 1:5 (heat shocked) serial dilution of randomly chosen and pooled samples, and *hsp70* values were normalized to β*-actin* to obtain relative quantities. The suitability of β*-actin* as a consistent house-keeping reference gene was determined using BestKeeper (Pfaffl et al., 2004). Because of the long stability and large amount of previously produced β*-actin* mRNA, transcript amounts remained unchanged throughout the study, even though it is likely that the formation of new mRNA varies during the experiment. In conclusion, the results give the relative quantities as the ratio between *hsp70* and β*-actin* mRNA levels.

### Western blotting for hsp70 determination

Frozen tissues were weighed and homogenized in 5 volumes of lysis buffer (62.5 mM Tris-HCl, 1μg/ml leupeptin, pepstatin, antipain and 1mM PMSF) using a TissueLyser (Qiagen, USA) at 30 shakes/s for 2 min. Lysates were kept on ice for 30 min prior to +4°C centrifugation at 10,000 RCF for 30 min and supernatant storage at −80°C. Protein concentrations were determined using the Bradford method (Bradford, 1976) and a protein assay dye reagent (Bio-Rad, Germany), with a serial dilution of bovine serum albumin (1 mg/ml) as a standard. Spectrophotometric measurements were performed at 595 nm using a Wallac EnVision 2103 Multilabel Reader (PerkinElmer, Finland).

An equal amount (20 μg) of protein per sample was mixed with 5X Laemmli buffer (Laemmli, 1970) and denatured for 5 min at 95°C, then loaded onto an SDS-PAGE gel comprised of 10% polyacrylamide. Gels were placed in a Mini-Protean 3 electrophoresis module (Bio-Rad, USA) and the proteins separated by size, first at 100 V for 30 min then 150 V for 1 h. Proteins were transferred onto a nitrocellulose membrane (Perkin Elmer, USA) at 100 V for 1 h at +4°C and incubated in PBS blocking solution containing 3% non-fat powdered milk and 0.3% Tween for 1 h. Membranes were incubated overnight simultaneously with rabbit polyclonal anti-salmonid inducible hsp70 (AS05061A) primary antibody (1:10000) (Agrisera, Sweden), and rabbit polyclonal anti-β-actin (ab8227) primary antibody (1:5000) (Abcam, UK) in PBS-Tween with 3% milk at + 4°C. Thereafter, membranes were incubated in PBS-Tween with 3% milk with HRP-conjugated anti-rabbit secondary antibody (1:2500) (Sigma-Aldrich, USA) for 1 h at room temperature, then washed and immersed in Amersham ECL Prime Western Blotting Detection Reagent (GE Healthcare, UK), followed by exposure to x-ray film. A short exposure (~5 s) for β-actin and a longer exposure (~2 min) for hsp70 was used to acquire a quantifiable signal in undisturbed fish, while a short exposure (~5 s) was used for both β-actin and hsp70 for heat shocked fish. Thus, the data do not allow the absolute levels of the two proteins to be compared, but since all the experimental time points were treated similarly, the data enable normalization. Densitometry was performed using ImageJ 1.48v (NIH, USA) and relative quantities were obtained by normalizing hsp70 values to β-actin. The levels of β-actin did not change significantly over time and between treatments in both species, thus confirming its suitability as a loading control and reference protein. Consequently, the results give the relative quantities as the ratio between hsp70 and β-actin bands.

### Statistics

**Figure 1:**
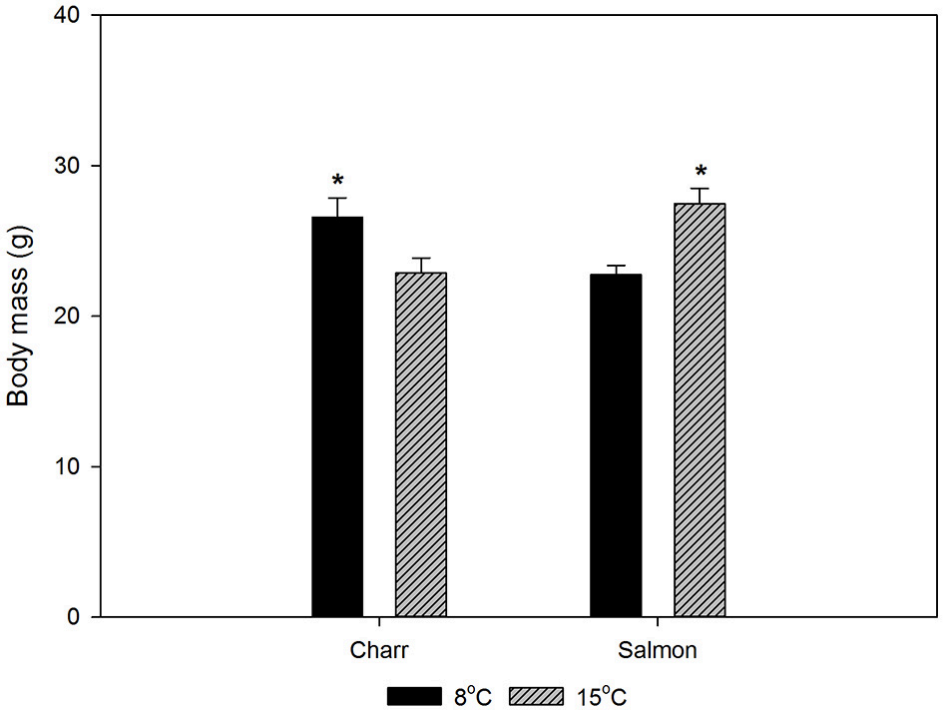
**The body masses of Arctic charr and Atlantic salmon after 1-month acclimation to 8 or 15°C**. Before the period of acclimation the fish in each species were held in one patch, so that any differences reflect the effects of acclimation period. Throughout acclimation the fish were fed daily *ad libitum*. The statistical significance of the difference in weight between 8 and 15°C-acclimated fish was tested with *t*-test. *p* < 0.05 was accepted as a statistically significant effect, indicated with ^*^ in the figure, mean ± SEM; *N* = 100.

**Figure 2:**
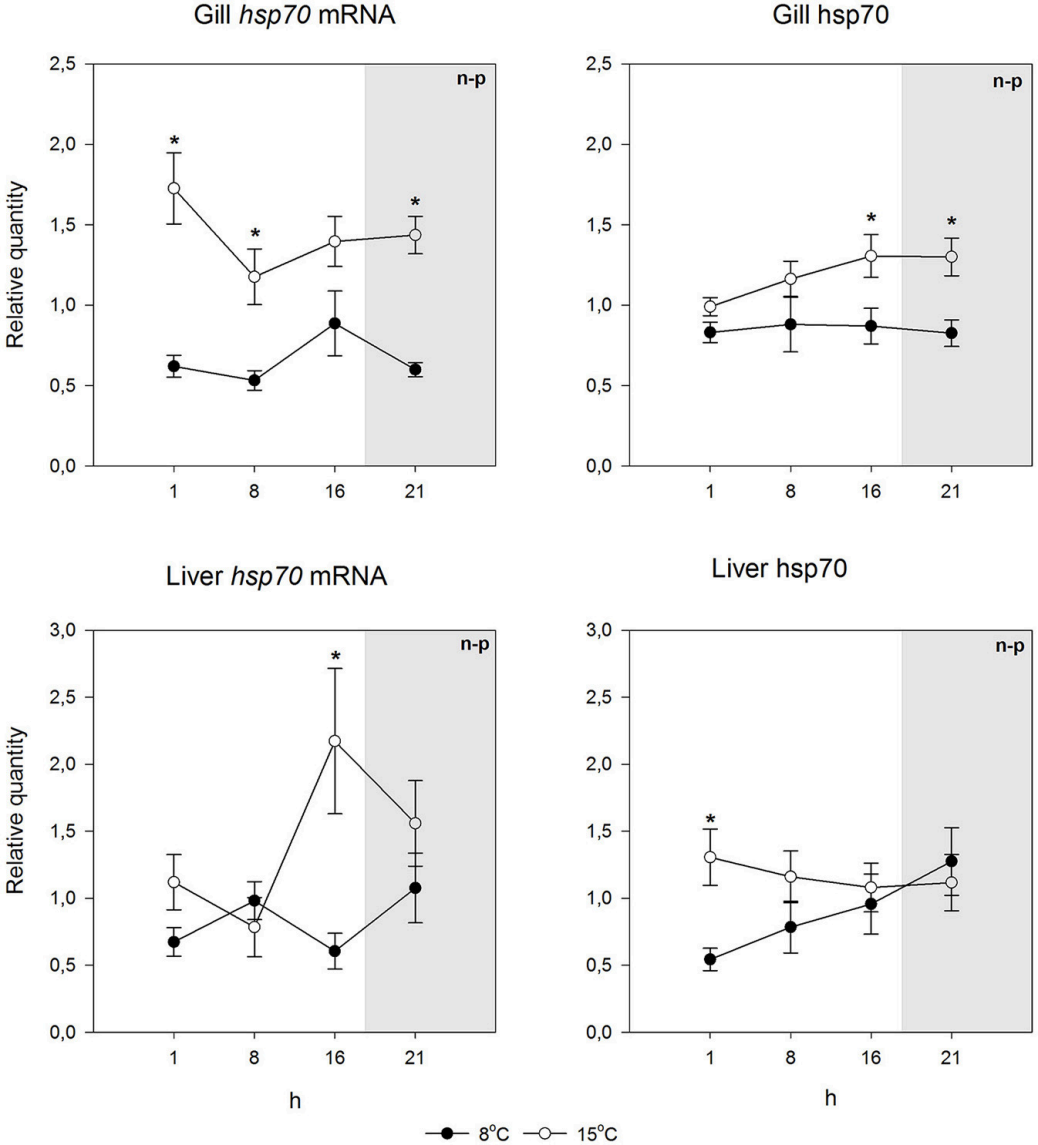
*****Hsp70*** mRNA and protein levels at different times of the 24-h light/dark cycle in undisturbed Arctic charr**. Relative quantities (mean ± SEM; *n* = 7) are given. Whenever ANOVA or Kruskal-Wallis on ranks [parametric (p) or non-parametric (n-p), respectively] indicated statistically significant differences *post-hoc* testing (Holm-Sidak or Dunn's method) was conducted. ^*^ indicates significant differences between acclimation temperatures at the same time point (*p* < 0.05 was accepted as statistically significant effect). There were no significant differences between time points within an acclimation temperature. Dark period is indicated by gray shading.

**Figure 3:**
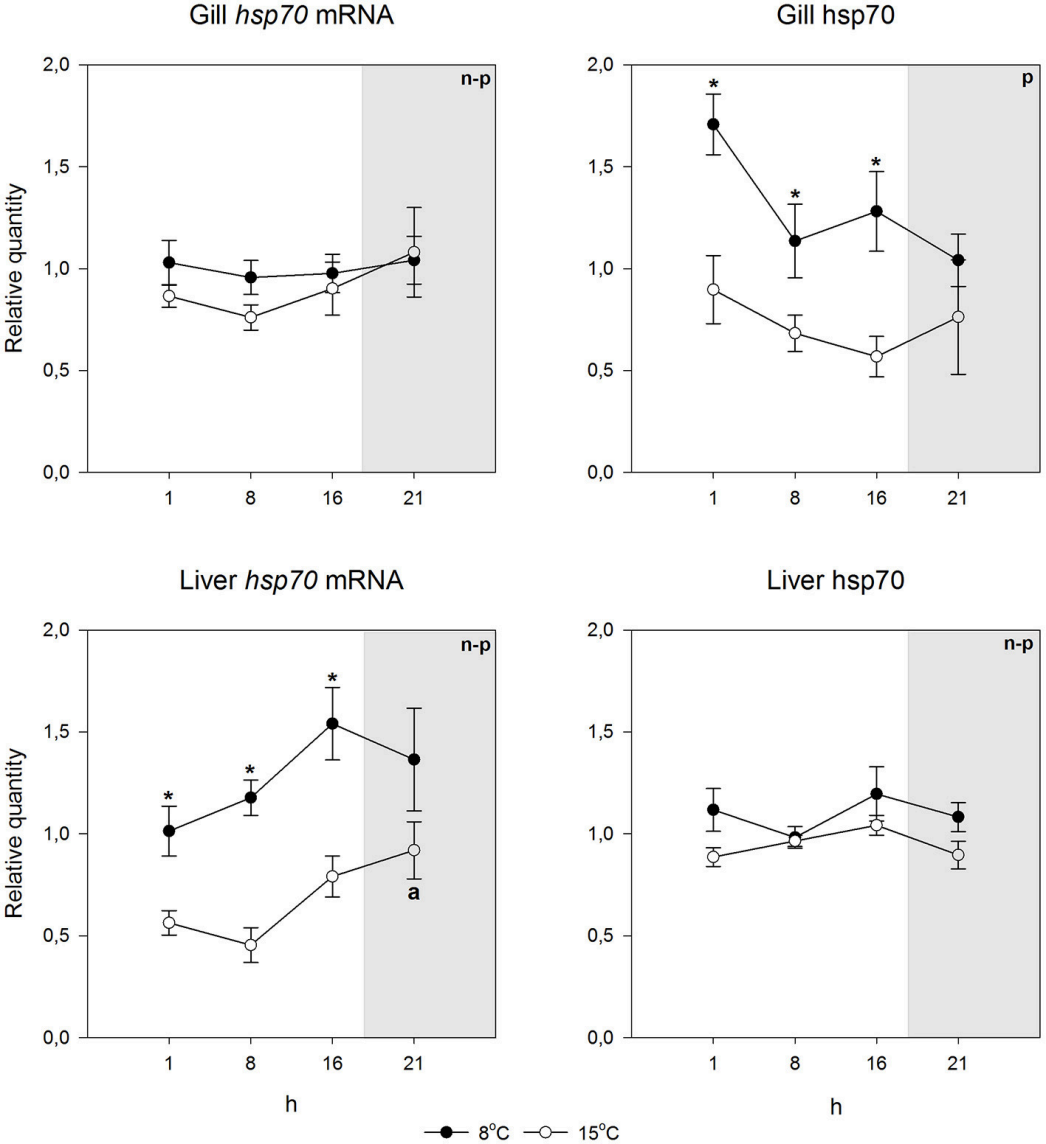
*****Hsp70*** mRNA and protein levels at different times of the 24-h light/dark cycle in undisturbed Atlantic salmon**. Relative quantities (mean ± SEM; *n* = 7) are given. Whenever ANOVA or Kruskal-Wallis on ranks [parametric (p) or non-parametric (n-p), respectively] indicated statistically significant differences *post-hoc* testing (Holm-Sidak or Dunn's method) was conducted. ^*^indicates significant differences between acclimation temperatures at the same time point, and different letters indicate that the means in those time points differ from each other (*p* < 0.05 was accepted as statistically significant effect). Dark period is indicated by gray shading.

It was initially tested if our data were normally distributed (Shapiro-Wilk's test) and had equal variances between groups (Brown-Forsythe's test). Since the data were in most cases not normally distributed, we first tried simple data transformations (e.g., log transformation) to make the data normal. However, this was not the case even after the transformation for most groups of data. This precludes using multivariate ANOVAs, which require normal distribution. Consequently, either parametric ANOVA or non-parametric Kruskal-Wallis test on ranks was used on mRNA and protein levels separately, with either acclimation temperature or time as an independent factor. We followed the suggested *post-hoc* testing given by Sigmaplot 13 (Holm-Sidak test for ANOVA, Dunn's test for Kruskal-Wallis on ranks [[**Figures 2, 3**]] or Dunnett's [**Figures 5, 6**]) whenever significant effects were identified. In Figure 1 the weights of the fish of each species were separately compared at 8 and 15°C using *t*-test. Since no changes occurred as a result of the 7°C temperature increase in cold-acclimated specimens, the effect of time in the heat shock experiments was only tested in warm-acclimated animals (There was one exception to this generalization; the hsp70 level in salmon gills was significantly higher prior to heat shock than at subsequent time points in cold-acclimated specimens). SigmaPlot 13 (SyStat Software, USA) was used for statistical comparisons and *p* < 0.05 was accepted to indicate a statistically significant effect.

## Results

For the studies, we acclimated Arctic charr and salmon to 8 and 15°C for 4 weeks. Figure 1 gives the weights of the fish after acclimation. Eight-degree-acclimated charr were heavier than those acclimated to 15°C, whereas the opposite was true for salmon. However, Fulton's condition factor (*K* = 100 × weight/length^3^) was essentially independent of the acclimation temperature with values of 0.809 ± 0.2 and 0.808 ± 0.01 (SEM) for 8 and 15°C-acclimated charr, and 1.064 ± 0.01 and 1.060 ± 0.01 for 8 and 15°C-acclimated salmon (*N* = 100), respectively.

**Figure 4:**
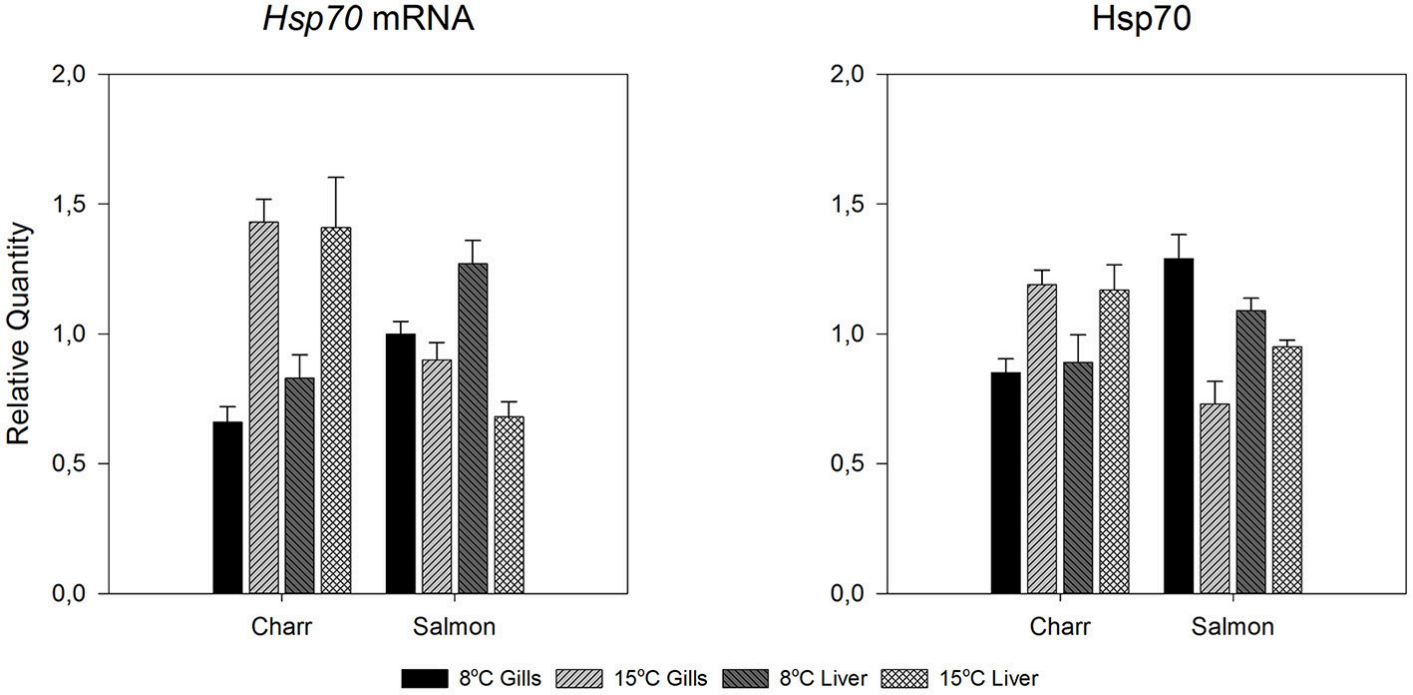
*****Hsp70*** mRNA and protein levels of undisturbed Arctic charr and Atlantic salmon after 1-month acclimation to 8 or 15°C in gills and liver**. Relative quantities are given. The figure reproduces data of Figures 2, 3 for giving the reader a clear picture how *hsp70* mRNA and protein changes between acclimation temperatures in the two species and tissues (gills or liver).

Using species-specific *inducible hsp70* primers and an antibody recognizing the inducible hsp70 in both species, for qPCR and immunoblotting, respectively, we first checked if the constitutive mRNA and protein expression varied during the day. This was deemed to be important, as light rhythm variations in the Arctic are pronounced, and light-temperature relationship will change as a consequence of climate change. Further, studies by Healy and Schulte (2012) have shown that *hsp70* level can show circadian fluctuations in *Fundulus heteroclitus*. Figures 2, 3 indicate that neither the *hsp70* mRNA nor the protein levels showed strong circadian fluctuations in either species, temperature or tissue (liver or gills) with the used light rhythm and sampling protocol. The exception to this generalization is the mRNA level in warm-acclimated salmon liver (*H*_3_ = 11.339, *p* = 0.01). However, the results show increased *hsp70* mRNA (*H*_1_ = 29.598, *p* < 0.001 and *H*_1_ = 7.222, *p* = 0.007 for gills and liver, respectively) and protein levels (*H*_1_ = 15.726, *p* < 0.001 and *H*_1_ = 5.491, *p* = 0.019 for gills and liver, respectively) in 15°C-acclimated charr as compared to 8°C-acclimated charr (Figures 2, 4). In contrast, in salmon the *hsp70* mRNA (*H*_1_ = 4.129, *p* = 0.042 and *H*_1_ = 23.846, *p* < 0.001 for gills and liver, respectively) and protein levels (*F*_1_ = 19.921, *p* < 0.001 and *H*_1_ = 5.962, *p* = 0.015 for gills and liver, respectively) were higher at the lower than at the higher acclimation temperature (Figures 3, 4). Further, it is possible that the mRNA-protein expression relationship is different in the two tissues in salmon.

**Figure 5:**
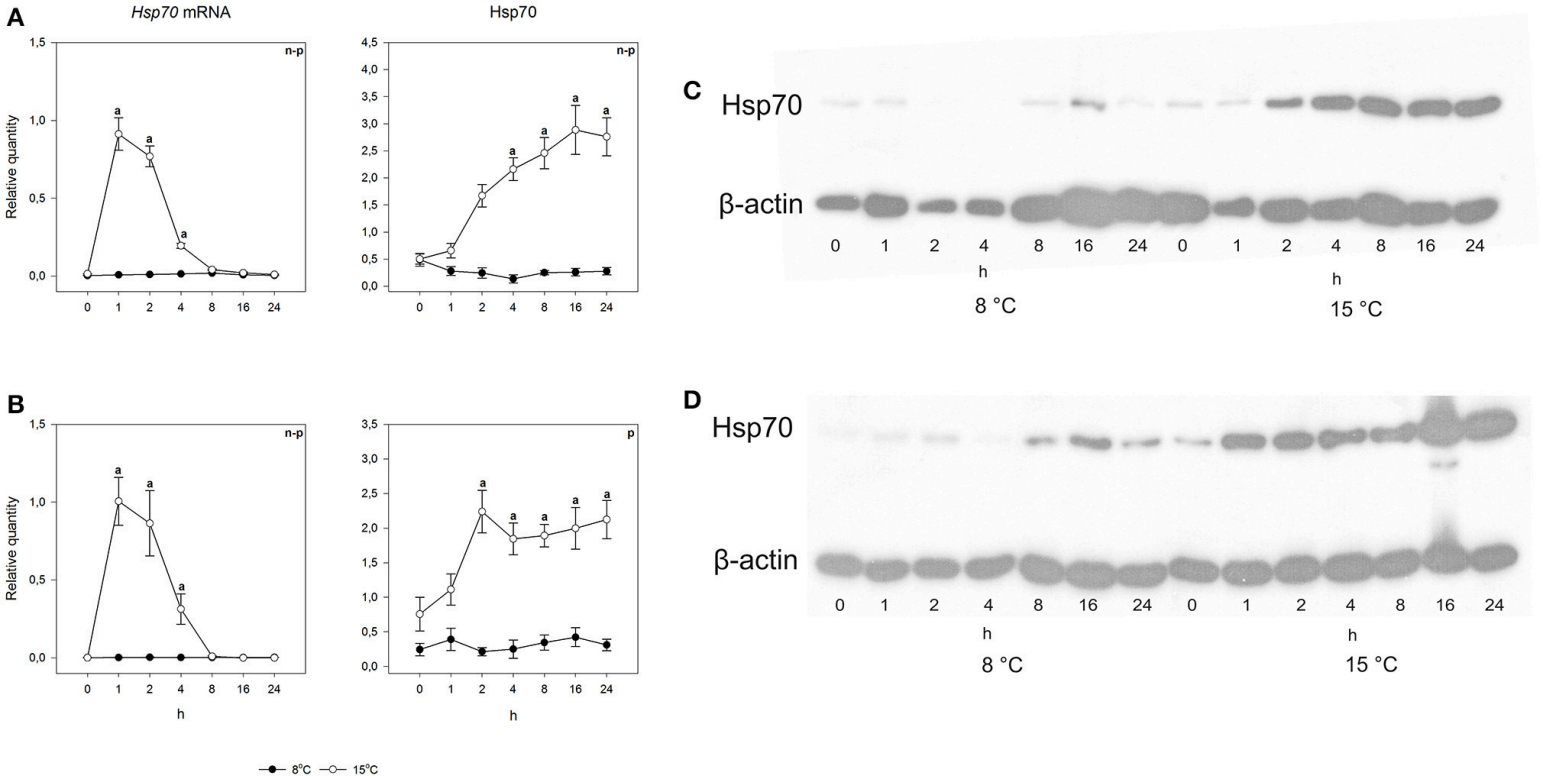
**Time course of heat shock induced ***hsp70*** mRNA and protein synthesis in Arctic charr**. Relative quantities (mean ± SEM; *n* = 7) in **(A)** gills and **(B)** liver. Relative quantities of hsp70 are based on hsp/β-actin ratios in the western blots. Representative examples of western blots for gills are given in **(C)** and liver in **(D)**. Whenever ANOVA or Kruskal-Wallis on ranks [parametric (p) or non-parametric (n-p), respectively] indicated statistically significant differences *post-hoc* testing (Dunnett's method) was conducted. A letter above a symbol indicates that the levels at that time point are significantly different from levels before the heat shock (*p* < 0.05).

**Figure 6:**
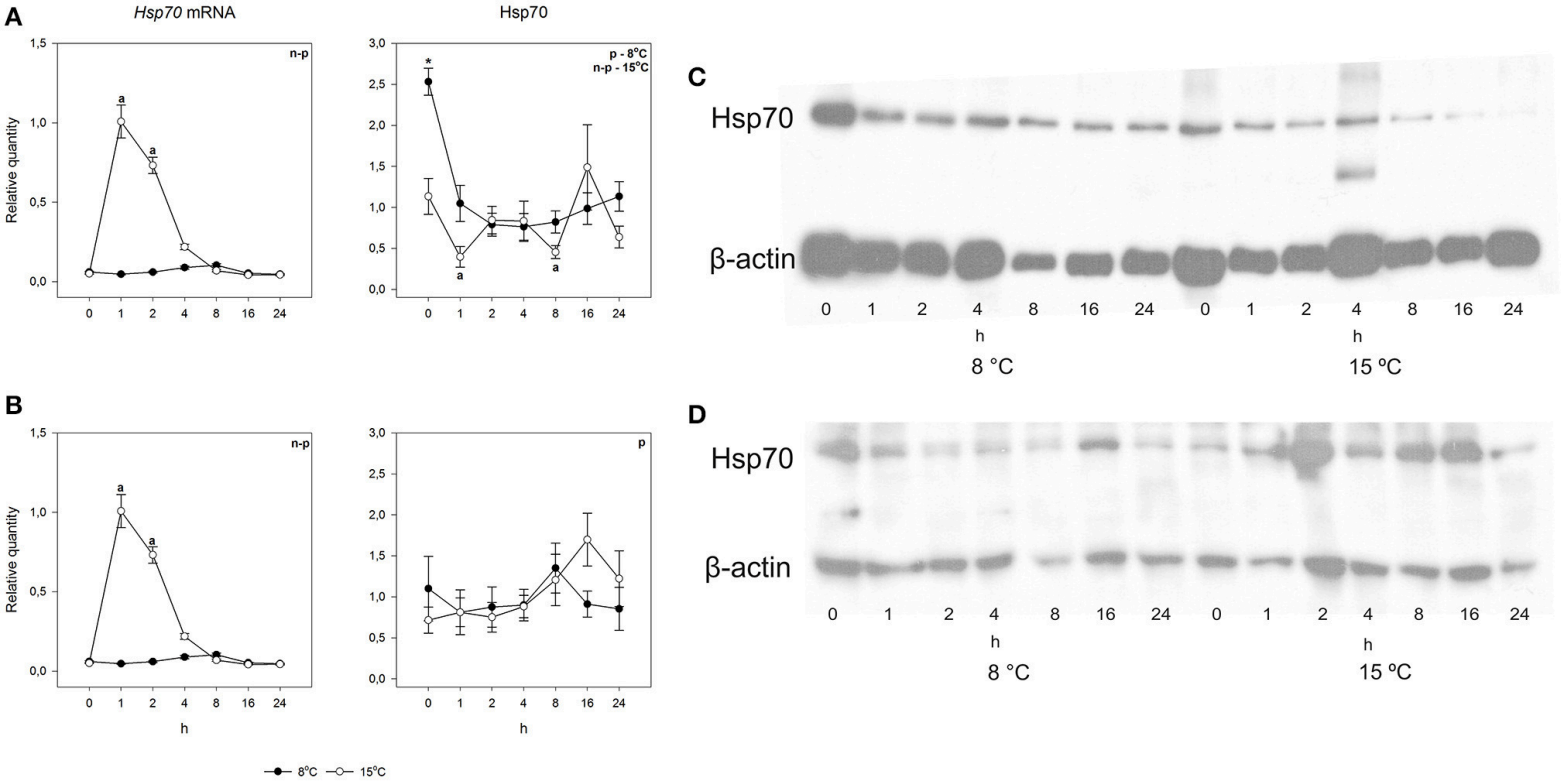
**Time course of heat shock induced ***hsp70*** mRNA and protein synthesis in Atlantic salmon**. Relative quantities (mean ± SEM, *n* = 7) in **(A)** gills and **(B)** liver. Relative quantities of hsp70 are based on hsp/β-actin ratios in the western blots. Representative examples of western blots for gills are given in **(C)** and liver in **(D)**. Whenever ANOVA or Kruskal-Wallis on ranks [parametric (p) or non-parametric (n-p), respectively] indicated statistically significant differences *post-hoc* testing (Dunnett's method) was conducted. A letter above a symbol indicates that the values at that time point are significantly different from the values before the heat shock. ^*^above the symbol in the hsp70 data of gills indicates the one significant difference found in cold-acclimated fish (*p* < 0.05).

To study the heat shock response, fish were exposed for 30 min to a temperature 7°C higher than the acclimation temperature, whereafter they were returned and the responses followed at the acclimation temperature. An acute increase in temperature from 8 to 15°C did not cause changes in either species in either mRNA or protein levels (Figures 5, 6). However, when the temperature increase was from 15 to 22°C, *hsp70* mRNA increased drastically in both species. Pre-exposure values were restored by 8 h after the fish were returned to the acclimation temperature. The rapid temperature-dependent transcriptional induction is the hallmark of the heat shock response (Lindquist, 1986). In contrast, the heat shock protein response was markedly different in the two species. Arctic charr showed the traditional pattern, where transcriptional induction was followed by protein production (Figure 5). The speed of protein accumulation was markedly different in liver and gills. Thus, in liver, the major detoxifying tissue (Hinton et al., 2008), the highest hsp70 level was reached already 2 h after the heat shock, whereas in gills the highest protein level was seen after 16 h (Figure 5). Conversely, in the 15°C-acclimated salmon subjected to a 7°C temperature increase hsp70 did not accumulate despite transcriptional induction (Figure 6).

## Discussion

The major finding of the present study was that despite the increase of *hsp70* mRNA the protein level did not increase in the 15°C-acclimated salmon. This result indicates that there are conditions when the notion that an increase in *hsp* mRNA indicates that also hsp increases in fish does not hold, a finding extending from those of Lund et al. (2002) and Hofmann et al. (2005). Earlier, it has been shown for *Xenopus* oocytes that the production of *hsp* mRNA and protein are uncoupled (Bienz and Gurdon, 1982), the heat shock response being regulated translationally (the heat shock response involves an increase in heat shock protein level but no change in mRNA). Our result gives a new dimension to the overall regulation of the heat shock response. While in both charr and salmon the heat shock gene is clearly transcriptionally regulated, as shown by the increase in mRNA in both species, the hsp level need not increase, as the result with salmon indicates. Naturally, our results are restricted to the induction time (30 min), and the following follow-up time (24 h). We cannot be certain that increasing the length of either would not be seen as increased protein production in salmon. However, we consider it improbable that increasing the follow-up time would have resulted in increased hsp70 level, as a significant protein level change occurred in 2 h in charr liver but not after 24 h in salmon liver. In contrast, it is possible that increasing the length of exposure could have caused the salmon hsp70 level to increase, as the signal for hsp accumulation is most likely the amount of misfolded protein, which increases with time.

The above conclusion also depends critically on whether the protein recognized by the antibody is inducible in both charr and salmon. This is most probable as the antibody used has earlier successfully been used to probe inducible hsp70 level in salmon (Tunnah et al., 2016), where the increase of protein level was not observed in the present study. In addition, the antibody has successfully been used to document hsp70 induction in the central mudminnow (*Umbra limi*) (Currie et al., 2010). It should be noted that at least in insects the production of heat shock protein is related to the steady-state (resting) level of the protein: if the resting level is high, hsp production may not take place (Zatsepina et al., 2016).

The question is then why there should be such a prevention of heat shock protein production. The reason may be related to the fact that during heat shock only heat shock proteins are translated, with their preferential translation going on upon recovery from heat shock (Storti et al., 1980). The translation of other proteins gradually increases during recovery. Depending on the severity of shock the heat shock protein production can be short-term or sustained (Gedamu et al., 1983). If only heat shock proteins can be produced instead of other needed proteins, a serious cost is incurred. Such a cost would not take place if the production of heat shock proteins did not occur. The following two reasons have been earlier suggested as possible reasons why heat shock proteins are not always produced abundantly: first, in large amounts hsps might disturb the normal cellular/organismal functions, or, second, the production and degradation of hsps could cause intolerable increase in cellular energy consumption (Feder and Hofmann, 1999). The fact that the heat shock response is often absent in early development of organisms with otherwise pronounced protein synthesis (reviewed in Feder and Hofmann, 1999), (e.g., transcriptional induction of *hsp70* gene does not occur in early development of *Xenopus*; Heikkila et al., 1987), suggests that the competition for translation may be a significant reason for preventing heat shock protein production.

Our results add to the possibilities of regulating the heat shock response utilizing hsp70 at different levels. First, the transcriptional induction temperature of the genes encoding heat shock proteins differs between species and populations (Feder and Hofmann, 1999; Buckley and Hofmann, 2004), and is also affected by the acclimation temperature of the organisms (Tomanek and Somero, 1999, 2002; Podrabsky and Somero, 2004) with the complete lack of induction in some stenothermal organisms (Tomanek, 2010). Second, there are clearly several different proteins in the hsp70 family, which may have different transcriptional induction temperatures. Such a situation can be the basis of population differences in the induction of the response (Fangue et al., 2006). Our finding shows that the heat shock protein synthesis can also be controlled post-transcriptionally in fish. Here one has to note that the work was done very close to the temperature where the mRNA induction in Atlantic salmon is observed (see Lund et al., 2002), but much above the temperature required for mRNA accumulation in charr (Quinn et al., 2011). Consequently, the results cannot indicate what the temperature difference between having both the *hsp70* mRNA and protein accumulate, or having only the mRNA level to increase is.

The results thus indicate an obvious set of future experiments: carrying out acclimation of a species in a set of temperatures with consequent temperature increases of different magnitudes and different lengths. Based on our results we predict that (1) a given increase in temperature causes neither transcriptional nor translational induction of the heat shock gene at low acclimation temperature. (2) With an increase in temperature, the heat shock genes are first induced transcriptionally but not translationally. (3) When the acclimation temperature is high enough, both transcriptional and translational induction occur. The temperature difference between possibilities 2 and 3 is very interesting, as it affects the significance and use of the response. If the difference is species-dependent, large in some and small in others, a significant importance to it being an important step in the regulation of the heat shock response can be attached. If the temperature difference between 2 and 3 is narrow in all species, then the response can be used to probe if small increases in environmental temperatures have an effect on fish.

The results of the present study also show a clear time lag between transcriptional induction and protein production. The time lag has been experimentally shown (Buckley et al., 2006), but is inadequately characterized and taken into account (Logan and Buckley, 2015). For example, with circadian changes of protein levels the relevant transcription must take place several hours before the maximal amount of protein is required. This means that the cue for increased transcription cannot be the same as the reason for maximal protein level in the circadian cycle. Our results also indicate that the time lag between transcription and translation is, not surprisingly, cell type-specific. The simplest explanation for this is that the availability of ribosomes is the limiting factor and the time lag is shortest in cells with high probability of inducible protein production such as hepatocytes with their inducible detoxification machinery (Hinton et al., 2008). Further, the type of translated protein will affect the time lag. With regard to inducible heat shock proteins, the time lag is exceptionally short, which was thought to be due to them lacking introns (Molina et al., 2000). However, although mammalian and Drosophila *hsp* genes lack introns, they are present in fish genes (Metzger et al., 2016).

In an attempt of explaining the difference between the responses of the two species, the body mass data are useful. Since temperature did not affect the condition factor of either species significantly, the weight change reflects the overall growth. An increase in temperature initially increases the growth rate of fish until the optimum temperature (for growth) is reached, whereafter it decreases with temperature increase (Wootton, 2011). Since the charr from a single batch were smaller after acclimation to the higher than to the lower temperature, the higher acclimation temperature has been above the optimum temperature of charr. In contrast, the higher acclimation temperature has not exceeded, or at most only slightly exceeded the optimum temperature of salmon. Thus, the Arctic charr acclimated to 15°C are closer to the higher end of their temperature tolerance than the Atlantic salmon. This observation fits with earlier conclusions of the lower temperature tolerance of Arctic charr than salmon (Elliott and Elliott, 2010). The difference is reflected in the level of both *hsp* mRNA and protein in undisturbed fish: in charr both were higher at 15°C than at 8°C, whereas in salmon the opposite was true. The situation in salmon is as expected from the effects of temperature on mRNA and protein breakdown, whereas that in charr likely represents suboptimal temperature. Notably, in practical aquaculture in the present hatchery, adult Arctic charr in the hatchery population start to show increased mortality when the temperature rises above 15°C.

Associated with the increased level of *hsp70* mRNA and protein in 15°C-acclimated, undisturbed fish, the charr showed the classical heat shock response with accumulation of both *hsp* mRNA and protein, whereas in salmon with no increase in the level of *hsp70* mRNA and protein in 15°C-acclimated, undisturbed fish, the accumulation of protein did not occur. While the present study was not designed to elucidate the mechanism of post-transcriptional regulation of the heat shock response, the result indicates an important role for it. Speculatively, when the temperature has increased adequately to cause transcriptional induction, but the change is not life-threatening, the mRNA is not translated to protein to prevent the high energy costs of translation (Schwanhäusser et al., 2011). It is possible that microRNAs are involved. An increase in temperature influences the formation of microRNAs (Yin et al., 2009), which block translation. Earlier, an untranslated region of human *hsp70* mRNA has been shown to affect translational efficiency (Vivinus et al., 2001). If the *hsp70* mRNA is prevented from occupying ribosomes, they are available for translation of other proteins required for successful life at elevated temperature. One mechanism for post-transcriptional regulation of the HSR involves the temperature-induced decrease in the stability of *hsp70* mRNA (Theodorakis and Morimoto, 1987). In our case, this would result in some protein production in liver with its shorter time lag between transcription and translation than those of gills. A hint toward this is seen; the liver hsp70 level of the 15°C-acclimated salmon (Figure 6B) tended to become elevated after the heat shock. Another possibility is that the mRNA of *hsp70* plays a role in signaling.

In conclusion, the present results suggest that hsp70 production is prevented post-transcriptionally, when the temperature increases enough to cause transcriptional induction, but is not life threatening. Since the present studies were done close to the temperature required to see an mRNA response in Atlantic salmon, further studies are needed to evaluate the temperature difference between transcriptional induction and hsp70 accumulation in different species, and the mechanism by which translation is prevented. With regard to tools studying how critical a temperature increase is to survival of a species, both *hsp70* mRNA and protein should be quantitatively measured: if our prediction is right, an increase of both indicates that the species is at the high end of its tolerable temperature window. If only mRNA increases, the species is disturbed at the temperature studied, but can utilize post-transcriptional regulatory mechanisms to avoid the energy-costly translational response (Schwanhäusser et al., 2011).

## Author contributions

MN conceived the study and MN, ML, and KA designed the experiments. ML, KA, MK, MG, JP, ES, and IK performed the acclimation set-up, experiments and sampling. ML and MG conducted the laboratory experiments and ML and MN performed the data and statistical analyses. MN and ML drafted the manuscript and all authors contributed in the revision and gave final approval for publication.

## Funding

Suomen Akatemia, Biotieteiden ja Ympäristön Tutkimuksen Toimikunta (258078, MN); European Commission, Seventh Framework Programme, Marie Curie Actions (623338, MG); Koneen säätiö (KA); Doctoral Programme of Biological Interactions (JP).

### Conflict of interest statement

The authors declare that the research was conducted in the absence of any commercial or financial relationships that could be construed as a potential conflict of interest. The reviewer SF and handling Editor declared their shared affiliation, and the handling Editor states that the process nevertheless met the standards of a fair and objective review.

## References

[B1] Anttila K. Lewis M. Prokkola J. M. Kanerva M. Seppänen E. Kolari I.. (2015). Warm acclimation and oxygen depletion induce species-specific responses in salmonids. J. Exp. Biol. 218, 1471–1477. 10.1242/jeb.119115 25827840

[B2] Aranda P. S. LaJoie D. M. Jorcyk C. L. (2012). Bleach gel: a simple agarose gel for analyzing RNA quality. Electrophoresis 33, 366–369. 10.1002/elps.201100335 22222980 PMC3699176

[B3] Basu N. Todgham A. E. Ackerman P. A. Bibeau M. R. Nakano K. Schulte P. M.. (2002). Heat shock protein genes and their functional significance in fish. Gene 295, 173–183. 10.1016/S0378-1119(02)00687-X 12354651

[B4] Belkin I. M. (2004). Rapid warming of large marine ecosystems. Prog. Oceanogr. 81, 207–213. 10.1016/j.pocean.2009.04.011

[B5] Bienz M. Gurdon J. B. (1982). The heat-shock response in *Xenopus* oocytes is controlled at the translational level. Cell 29, 811–819. 10.1016/0092-8674(82)90443-3 6891290

[B6] Bradford M. M. (1976). A rapid and sensitive method for the quantitation of microgram quantities of protein utilizing the principle of protein-dye binding. Anal. Biochem. 72, 248–254. 10.1016/0003-2697(76)90527-3 942051

[B7] Buckley B. A. Gracey A. Y. Somero G. N. (2006). The cellular response to heat stress in the goby *Gillichthys mirabilis*: a cDNA microarray and protein-level analysis. J. Exp. Biol. 209, 2660–2677. 10.1242/jeb.02292 16809457

[B8] Buckley B. A. Hofmann G. E. (2004) Magnitude duration of thermal stress determine kinetics of hsp gene regulation in the goby *Gillichthys mirabilis*. Physiol. Biochem. Zool. 77, 570–581. 10.1086/420944 15449228

[B9] Chomczynski P. Sacchi N. (1987). Single-step method of RNA isolation by acid guanidinium thiocyanate-phenol-chloroform extraction. Anal. Biochem. 162, 156–159. 10.1016/0003-2697(87)90021-2 2440339

[B10] Currie S. Leblanc D. M. Ong K. J. (2010). Metabolism, nitrogen excretion and heat shock proteins in the central mudminnow (*Umbra limi*), a facultative air-breathing fish living in a variable environment. Can. J. Zool. 88, 43–58. 10.1139/Z09-117

[B11] Deane E. E. Woo N. Y. S. (2011). Advances and perspectives on the regulation and expression of piscine heat shock proteins. Rev. Fish Biol. Fish 21, 153–185. 10.1007/s11160-010-9164-8

[B12] Dietz T. J. Somero G. N. (1992). The threshold induction temperature of the 90-kDa heat shock protein is subject to acclimatization in eurythermal goby fishes (genus *Gillichthys*). Proc. Natl. Acad. Sci. U.S.A. 89, 3389–3393. 1565632 10.1073/pnas.89.8.3389 PMC48873

[B13] Elliott J. M. Elliott J. A. (2010). Temperature requirements of Atlantic salmon *Salmo salar*, brown trout *Salmo trutta* and Arctic charr *Salvelinus alpinus*: predicting the effects of climate change. J. Fish Biol. 77, 1793–1817. 10.1111/j.1095-8649.2010.02762.x 21078091

[B14] Fangue N. A. Hofmeister M. Schulte P. M. (2006). Intraspecific variation in thermal tolerance and heat shock protein gene expression in common killifish, *Fundulus heteroclitus*. J. Exp. Biol. 209, 2859–2872. 10.1242/jeb.02260 16857869

[B15] Feder M. E. Hofmann G. E. (1999). Heat-shock proteins, molecular chaperones, and the stress response: evolutionary and ecological physiology. Annu. Rev. Physiol. 61, 243–282. 10.1146/annurev.physiol.61.1.243 10099689

[B16] Gedamu L. Culham B. Heikkila J. J. (1983). Analysis of the temperature-dependent temporal pattern of heat-shock-protein synthesis in fish cells. Biosci. Rep. 3, 647–658. 10.1007/BF01172875 6626707

[B17] Healy T. M. Schulte P. M. (2012). Factors affecting plasticity in whole-organism thermal tolerance in common killifish (*Fundulus heteroclitus*). J. Comp. Physio. B Biochem. Syst. Environ. Physiol. 182, 49–62. 10.1007/s00360-011-0595-x 21698526

[B18] Healy T. M. Tymchuk W. E. Osborne E. J. Schulte P. M. (2010). Heat shock response of killifish (*Fundulus heteroclitus*): candidate gene and heterologous microarray approaches. Physiol. Genomics 41, 171–184. 10.1152/physiolgenomics.00209.2009 20103695

[B19] Heikkila J. J. Ovsenek N. Krone P. (1987). Examination of heat shock protein mRNA accumulation in early *Xenopus laevis* embryos. Biochem. Cell Biol. 65, 87–94. 10.1139/o87-013 3828114

[B20] Hinton D. E. Segner H. Au D. W. T. Kullman S. W. Hardman R. C. (2008). Liver toxicity, in The Toxicology of Fishes, eds Di Giulio R. T. Hinton D. E. (Boca Raton, FL: CRC Press), 327–400. 10.1201/9780203647295.ch7

[B21] Hofmann G. E. Lund S. G. Place S. P. Whitmer A. C. (2005). Some like it hot, some like it cold: the heat shock response is found in New Zealand but not Antarctic notothenioid fishes. J. Exp. Mar. Biol. Ecol. 316, 79–89. 10.1016/j.jembe.2004.10.007

[B22] Jayapal K. P. Philp R. J. Kok Y. J. Yap M. G. Sherman D. H. Griffin T. J.. (2008). Uncovering genes with divergent mRNA-protein dynamics in *Streptomyces coelicolor*. PLoS ONE 3:e2097. 10.1371/journal.pone.0002097 18461186 PMC2367054

[B23] Kaarniranta K. Elo M. Sironen R. Lammi M. J. Goldring M. B. Eriksson J. E.. (1998). Hsp70 accumulation in chondrocytic cells exposed to high continuous hydrostatic pressure coincides with mRNA stabilization rather than transcriptional activation. Proc. Natl. Acad. Sci. U.S.A. 95, 2319–2324. 10.1073/pnas.95.5.2319 9482883 PMC19331

[B24] Kaarniranta K. Oksala N. Karjalainen H. M. Suuronen T. Sistonen L. Helminen H. J.. (2002). Neuronal cells show regulatory differences in the hsp70 gene response. Brain Res. 101, 136–140. 10.1016/S0169-328X(02)00179-1 12007842

[B25] Laemmli U. K. (1970). Cleavage of structural proteins during the assembly of the head of bacteriophage T4. Nature 227, 680–685. 10.1038/227680a0 5432063

[B26] Lindquist S. (1986). The heat-shock response. Annu. Rev. Biochem. 55, 1151–1191. 10.1146/annurev.bi.55.070186.005443 2427013

[B27] Lindquist S. Craig E. A. (1988). The heat-shock proteins. Annu. Rev. Genet. 22, 631–677. 10.1146/annurev.ge.22.120188.003215 2853609

[B28] Logan C. A. Buckley B. A. (2015). Transcriptomic responses to environmental temperature in eurythermal and stenothermal fishes. J. Exp. Biol. 218, 1915–1924. 10.1242/jeb.114397 26085668

[B29] Lund S. G. Caissie D. Cunjak R. A. Vijayan M. M. Tufts B. L. (2002). The effects of environmental heat stress on heat-shock mRNA and protein expression in Miramichi Atlantic salmon (*Salmo salar*) parr. Can. J. Fish. Aquat. Sci. 59, 1553–1562. 10.1139/f02-117

[B30] Melling C. W. Thorp D. B. Milne K. J. Krause M. P. Noble E. G. (2007). Exercise-mediated regulation of Hsp70 expression following aerobic exercise training. Am. J. Physiol. Heart Circ. Physiol. 293, H3692–H3698. 10.1152/ajpheart.00827.2007 17921326

[B31] Metzger D. C. H. Hemmer-Hansen J. Schulte P. M. (2016). Conserved structure and expression of *hsp70* paralogs in teleost fishes. Comp. Biochem. Physiol. D 18, 10–20. 10.1016/j.cbd.2016.01.007 26922644

[B32] Molina A. Biemar F. Muller F. Iyengar A. Prunet P. Maclean N.. (2000). Cloning and expression analysis of an inducible *HSP70* gene from tilapia fish. FEBS Lett. 474, 5–10. 10.1016/S0014-5793(00)01538-6 10828441

[B33] Morimoto R. I. (1993). Cells in stress: transcriptional activation of heat shock genes. Science 259, 1409–1410. 10.1126/science.8451637 8451637

[B34] Pfaffl M. W. Tichopad A. Prgomet C. Neuvians T. P. (2004). Determination of stable housekeeping genes, differentially regulated target genes and sample integrity: BestKeeper–Excel-based tool using pair-wise correlations. Biotechnol. Lett. 26, 509–515. 10.1023/B:BILE.0000019559.84305.47 15127793

[B35] Prahlad V. Morimoto R. I. (2009). Integrating the stress response: lessons for neurodegenerative diseases from *C. elegans*. Trends Cell Biol. 19, 52–61. 10.1016/j.tcb.2008.11.002 19112021 PMC4843516

[B36] Podrabsky J. E. Somero G. N. (2004). Changes in gene expression associated with acclimation to constant temperatures and fluctuating daily temperatures in an annual killifish *Austrofundulus limnaeus*. J. Exp. Biol. 207, 2237–2254. 10.1242/jeb.01016 15159429

[B37] Quinn N. L. McGowan C. R. Cooper G. A. Koop B. F. Davidson W. S. (2011). Ribosomal genes and heat shock proteins as putative markers for chronic, sublethal heat stress in Arctic charr: applications for aquaculture and wild fish. Physiol. Genomics 43, 1056–1064. 10.1152/physiolgenomics.00090.2011 21750231

[B38] Richter K. Haslbeck M. Buchner J. (2010). The heat shock response: life on the verge of death. Mol. Cell 40, 253–266. 10.1016/j.molcel.2010.10.006 20965420

[B39] Schwanhäusser B. Busse D. Li N. Dittmar G. Schuchhardt J. Wolf J.. (2011). Global quantification of mammalian gene expression control. Nature 473, 337–342. 10.1038/nature10098 21593866

[B40] Silver J. T. Noble E. G. (2012). Regulation of survival gene hsp70. Cell Stress Chaper. 17, 1–9. 10.1007/s12192-011-0290-6 21874533 PMC3227850

[B41] Sistonen L. Sarge K. D. Morimoto R. I. (1994). Human heat shock factors 1 and 2 are differentially activated and can synergistically induce hsp70 gene transcription. Mol. Cell. Biol. 14, 2087–2099. 10.1128/MCB.14.3.2087 8114740 PMC358569

[B42] Storti R. V. Scott M. P. Rich A. Pardue M. L. (1980). Translational control of protein synthesis in response to heat shock in *D. melanogaster* cells. Cell 22, 825–834. 10.1016/0092-8674(80)90559-0 6780199

[B43] Theodorakis N. G. Morimoto R. I. (1987). Posttranscriptional regulation of hsp70 expression in human cells: effects of heat shock, inhibition of protein synthesis, and adenovirus infection on translation and mRNA stability. Mol. Cell. Biol. 7, 4357–4368. 10.1128/MCB.7.12.4357 3437893 PMC368119

[B44] Tomanek L. (2010). Variation in the heat shock response and its implication for predicting the effect of global climate change on species' biogeographical distribution ranges and metabolic costs. J. Exp. Biol. 213, 971–979. 10.1242/jeb.038034 20190122

[B45] Tomanek L. Somero G. N. (1999). Evolutionary and acclimation-induced variation in the heat-shock responses of congeneric marine snails (genus *Tegula*) from different thermal habitats: Implications for limits of thermotolerance and biogeography. J. Exp. Biol. 202, 2925–2936. 10518474 10.1242/jeb.202.21.2925

[B46] Tomanek L. Somero G. N. (2002). Interspecific- and acclimation-induced variation in levels of heat-shock proteins 70 (hsp70) and 90 (hsp90) and heat-shock transcription factor-1 (HSF1) in congeneric marine snails (genus *Tegula*): implications for regulation of hsp gene expression. J. Exp. Biol. 205, 677–685. 11907057 10.1242/jeb.205.5.677

[B47] Tunnah L. Currie S. MacCormack T. J. (2016). Do prior diel thermal cycles influence the physiological response of Atlantic salmon (*Salmo salar*) to subsequent heat stress? Can. J. Fish. Aquat. Sci. 10.1139/cjfas-2016-0157. [Epub ahead of print].

[B48] Vijayan M. M. Pereira C. Forsyth R. B. Kennedy C. J. Iwama G. K. (1997). Handling stress does not affect the expression of hepatic heat shock protein 70 and conjugation enzymes in rainbow trout treated with beta-naphthoflavone. Life Sci. 61, 117–127. 10.1016/S0024-3205(97)00366-4 9217270

[B49] Vivinus S. Baulande S. van Zanten M. Campbell F. Topley P. Ellis J. H.. (2001). An element within the 5′ untranslated region of human Hsp70 mRNA which acts as a general enhancer of mRNA translation. Eur. J. Biochem. 268, 1908–1917. 10.1046/j.1432-1327.2001.02064.x 11277913

[B50] Wanishsakpong W. McNeil N. Notodiputro K. A. (2016). Trend and pattern classification of surface air temperature change in the Arctic region. Atmosph. Sci. Lett. 17, 378–383. 10.1002/asl.668

[B51] Wootton R. J. (2011). Energy utilization in growth. Growth: environmental effects, in Encyclopedia of Fish Physiology, ed Farrell A. P. (San Diego, CA: Academic Press), 1629–1635.

[B52] Yin C. Salloum F. N. Kukreja R. C. (2009). A novel role of microRNA in late preconditioning: upregulation of endothelial nitric oxide synthase and heat shock protein 70. Circ. Res. 104, 572–575. 10.1161/CIRCRESAHA.108.193250 19213952 PMC3359791

[B53] Zatsepina O. G. Przhiboro A. A. Yushenova I. A. Shilova V. Zelentsova E. S. Shostak N. G.. (2016). A *Drosophila* heat shock response represents an exception rather than a rule amongst Diptera species. Insect Mol. Biol. 25, 431–449. 10.1111/imb.12235 27089053

